# Associations among fear of childbirth, resilience and psychological distress in pregnant women: A response surface analysis and moderated mediation model

**DOI:** 10.3389/fpsyt.2022.1091042

**Published:** 2022-12-15

**Authors:** Xiaoxiao Mei, Ranran Mei, Yuling Liu, Xinqin Wang, Qianwen Chen, Youjin Lei, Zengjie Ye

**Affiliations:** ^1^School of Nursing, Guangzhou University of Chinese Medicine, Guangzhou, Guangdong, China; ^2^Affiliated Cancer Hospital and Institute of Guangzhou Medical University, Guangzhou, Guangdong, China; ^3^The First Affiliated Hospital, Guangzhou University of Chinese Medicine, Guangzhou, Guangdong, China

**Keywords:** fear of childbirth, resilience, psychological distress, dominance analysis, response surface analysis, moderated mediation analysis, pregnant women

## Abstract

**Introduction:**

Prenatal psychological distress is prevalent during pregnancy. This study aimed to estimate the associations among fear of childbirth, resilience and psychological distress.

**Methods:**

A total of 1,060 Chinese pregnant women were enrolled from Be Resilient to Postpartum Depression (ChiCTR2100048465) and the following instruments were administered to them: Childbirth Attitudes Questionnaire, Connor-Davidson Resilience Scale, Perceived Social Support Scale, General Self-Efficacy Scale, Adverse Childhood Experience scale and Hospital Anxiety and Depression Scale. A dominance, a response surface analysis and a moderated mediation analysis were performed.

**Results:**

In terms of psychological distress, resilience and fear of childbirth could explain 41.6% (0.148/0.356) and 33.1% (0.118/0.356), respectively. Pregnant women with high resilience-low fear of childbirth had significantly lower levels of psychological distress than those with low resilience-high fear of childbirth. The indirect effects of fear of childbirth on psychological distress through resilience was significantly (*B* = 0.054, 95% CI 0.038 to 0.070). The interactions between fear of childbirth and adverse childhood experiences (β = 0.114, 95% CI −0.002 to 0.231, *p* = 0.054) and between resilience and adverse childhood experiences (β = −0.118, 95% CI −0.222 to −0.012, *p* < 0.05) were significant.

**Conclusion:**

Resilience, fear of childbirth and adverse childhood experiences may be three important factors to psychological distress in Chinese pregnant women.

## Introduction

Depression and anxiety, termed here “psychological distress” are a health issue for pregnant women, that are associated with negative repercussions in the offspring and maternal health (1–3).

Psychological distress is globally reported for pregnant women due to all kinds of challenges and changes (4) and a recent meta-analysis reported that the pooled prevalence of anxiety and depression was 30.5% and 25.6%, respectively (5).

Previous studies have found that psychological distress are related to socio-demographic factors and psychological variables. In terms of socio-demographics, family economic status, education level, occupation, and pregnancy planning status have been confirmed to be significantly associated with psychological distress (6–8). Moreover, research has shown self-efficacy to be negatively correlated with fear of childbirth (FOC) and psychological distress but positively correlated with resilience (9–11). Meanwhile, it’s been confirmed that social support is essential for the development of resilience and can act as a buffer against FOC and psychological distress (12–15). However, FOC is a particularly important risk factor for psychological distress among these psychological variables. A cohort study of 545 participants found that pregnant women with a high degree of FOC are more likely to have elevated psychological distress (16).

Adverse childhood experiences (ACEs) are characterized by exposure to abuse, neglect, and family dysfunction (17). It has been widely proved that ACEs had long-term impact on health status later in life, such as substance use patterns, coronary heart disease, depressive symptoms, post-traumatic stress disorder and so on (18–21). For pregnant women, a wealth of existing evidence has indicated ACEs confer risk to psychological distress during pregnancy (22–24). Even though the literature on the effects of FOC and ACEs on maternal psychological distress is robust, researchers commonly ignore protective and positive factors, such as resilience.

Resilience is a dynamic process that represent one’s ability to adapt successfully to challenges and to thrive in the face of adversity (25, 26). Prior studies suggested that resilience has a protective effect on psychological distress during pregnancy (27–29). Furthermore, in a Chinese study involving 646 pregnant women has shown that resilience was negatively associated with FOC (30). Meanwhile, a recent research found that resilience served as a moderator between ACEs and psychological distress among pregnant women (22). However, the researches measuring resilience in Chinese pregnant women and related effects on psychological distress are still scarce. The potential relationship of FOC, resilience, ACEs and psychological distress has not been systematically examined among pregnant women in previous studies. Therefore, this study hypothesized that

(1) FOC and resilience would be significant predictors of psychological distress;
(2) Resilience mediated the association between FOC and psychological distress;
(3) ACEs may play a moderation role among FOC, resilience and psychological distress.

## Materials and methods

### Participants

One thousand and sixty pregnant women in our ongoing Be Resilient to Postpartum Depression (BRPD) cohort were recruited between January 2022 and April 2022. Fifty-eight were excluded due to missing questionnaires, resulting in a final sample of 1002 (response rate 94.5%). The inclusion criteria were: (1) more than 18 years old; (2) pregnancy confirmed by ultrasonography; (3) could communicate fluently in Mandarin; (4) willing to participate in this study. The exclusion criteria was: (1) women with mental illness; (2) plan to terminate pregnancy. Written consent was obtained before the formal investigation.

### Instruments

#### Demographics

Based on previous literature (6, 31), we collected demographics (age, academic degree, marital status, working status, monthly average income, place of residence) and pregnancy-related information (maternity type, pregnancy type, weeks of pregnancy).

#### Fear of childbirth

The Childbirth Attitudes Questionnaire (CAQ), used to evaluate fear related to childbirth, was developed by Lowe (32). The CAQ contains 16 items and 4 dimensions (baby-related, pain and injury-related, general and personal control-related, medical interventions and hospital care-related fears). The sum score ranges from 16 to 64, with higher scores indicating more severe FOC. The validity and reliability of the Chinese version of the CAQ has been validated in pregnant women in China (14, 33). The Cronbach’s alpha for CAQ in the present study was 0.950.

#### Resilience

The 25-item Connor-Davidson Resilience Scale (CD-RISC) was developed by Connor and Davidson (34) and a 10-item version was later shortened and validated by Campbell-Sills and Stein (35). The Chinese version of CD-RISC 10 (36) consists of 10 items and ranges from 0 to 40, with higher scores indicating higher resilience levels. This scale has been successfully administered in our previous studies (37–41). The Cronbach’s alpha in the present study was 0.921.

#### Perceived social support

The Perceived Social Support Scale (PSSS) was used to measure perceived support from family, friends, and others (42). It is a 7-Likert scale (1–7, definitely disagree to definitely agree) with high scores indicating a high level of social support. The Chinese version has been shown to have good reliability and validity in the maternal population (43, 44). The Cronbach’s alpha in the present study was 0.957.

#### Self-efficacy

The General Self-efficacy Scale (GSES) was developed by Zhang and Schwarzer (45), and the Chinese version has been proved to be reliable when employed in Chinese pregnant women (10, 11). The scale includes 10 items and is rated on a 4-point-Likert scale ranging from 10 to 40. A higher score indicates a higher degree of self-efficacy. The Cronbach’s alpha for GSES in the present study was 0.943.

#### Adverse childhood experiences

Adverse Childhood Experience scale (ACE) was used to measured adverse childhood experiences during the respondent’s first 18 years of life (46). It includes 10 items under three categories: household dysfunction (5 items), abuse (3 items) and neglect (2 items) and were binary (yes vs. no). We categorized adverse childhood experiences into dichotomous outcomes – not experienced (score = 0, coded as 0) and experienced (score ≥ 1, coded as 1). The Chinese version of the ACEs scale was translated by Dr. David Yeung (47) and has been widely used in ACE researches (48, 49).

#### Psychological distress

Hospital Anxiety and Depression Scale (HADS), developed by Zigmond and Snaith (50), was used to assess psychological distress levels, including two dimensions: anxiety (7 items) and depression (7 items). It employs a 4-point scoring method (0–3) yielding a total score range of 0-42, with higher scores representing a higher degree of psychological distress (51). The Chinese version of HADS has been approved to be reliable (52, 53). The Cronbach’s alpha for HADS in the present study was 0.816.

### Data analysis

First, descriptive analysis was used to describe the demographics and pregnancy-related information. In addition, Pearson’s correlation analysis was performed to determine the relationships among resilience, perceived social support, self-efficacy, fear of childbirth and psychological distress.

Second, dominance analysis was conducted in order to determine the relative importance of resilience, perceived social support, self-efficacy, and fear of childbirth on psychological distress in this study. In this analysis all the model predictors were compared to each other and ranked by their relative importance (54). Dominance analysis is based on estimating an △R^2^ value for all possible subset models, so we conducted 37 regression analyses to compare predictors’ weight.

Third, polynomial regression with moderated response surface analysis (RSA) was performed to test the congruent and discrepant effects of pregnant women’s resilience and fear of childbirth on their psychological distress level (55). This regression consists of ten predictors: (1) resilience, (2) FOC, (3) resilience *resilience, (4) resilience * FOC, (5) FOC * FOC ^2^, (6) ACE, (7) ACE * resilience, (8) ACE * FOC, (9) ACE * resilience * resilience, (10) ACE * resilience * FOC, (11) ACE * FOC * FOC.

Fourth, Harman’s one-factor model was adopted to estimate potential existence of the common method variance [CMV, (56)]. Then, the mediator role of resilience was evaluated between FOC and psychological distress through the PROCESS macro (Model 4) of SPSS.

Fifth, a moderation analysis was employed to examine the moderating role of ACE on the associations among fear of childbirth, resilience and psychological distress through the PROCESS macro (Model 5, 7, 14) of SPSS.

R 3.3.2 (57) and SPSS 26.0 (IBM Corp., Armonk, NY, United States) were used for all statistical analyses.

### Ethical considerations

The present study was part of BRPD (Registration number: ChiCTR2100048465) and was approved by the ethics review committee of the participating hospital (No: K-2022-024). The participants were reassured that their personal data would be kept confidentially and used for academic research anonymously.

## Results

### Sample characteristics

Among the 1002 women in the present study, the mean age was 29.59 years (SD = 4.16), and 95.1% of pregnant women were married. More than half (67.9%) were in employment status and one in ten had an unplanned pregnancy. Other details were demonstrated in Table 1.

**TABLE 1:** Demographic and relevant variables differences in scores of psychological distress.

Variables	M ± SD	Overall sample (*N* = 1002)	*P*-value
Age, M(SD)	8.02 ± 4.96	29.59(4.16)	0.009
Academic degree, n(%)			0.056
High school or less	8.49 ± 5.04	356(35.5%)	
Junior college degree	7.80 ± 4.74	366(36.5%)	
Bachelor or above	7.70 ± 5.11	280(28.0%)	
Marital status, n(%)			0.037
Married	7.92 ± 4.85	953(95.1%)	
Unmarried	9.92 ± 6.43	49(4.9%)	
Working status, n(%)			0.047
On-the-job	7.80 ± 7.84	680(67.9%)	
Non-working	8.48 ± 5.18	322(32.1%)	
Monthly average income, n(%)			0.029
≤4000 RMB	8.60 ± 4.87	260(25.9%)	
>4000 RMB	7.82 ± 4.98	568(74.1)	
Place of residence, n(%)			0.012
City or town	7.53 ± 4.93	397(39.6%)	
Countryside	8.34 ± 4.96	422(60.4%)	
Maternity type, n(%)			0.368
Multipara	7.87 ± 5.17	474(47.3%)	
Primipara	8.15 ± 4.76	528(52.7%)	
Pregnancy types, n(%)			0.004
Planned pregnancy	7.86 ± 4.90	896(89.4%)	
Unplanned pregnancy	9.33 ± 5.27	106(10.6%)	
Weeks of pregnancy, n(%)			0.405
≤13	8.06 ± 5.00	336(33.5%)	
14–27	7.77 ± 4.83	376(37.5%)	
≥28	8.29 ± 5.06	290(29.0%)	
Resilience, M(SD)	8.02 ± 4.96	26.95(7.11)	<0.001
Perceived social support, M(SD)	8.02 ± 4.96	66.47(11.50)	<0.001
Self-efficacy, M(SD)	8.02 ± 4.96	25.86(6.60)	<0.001
Fear of childbirth, M(SD)	8.02 ± 4.96	30.97 (10.20)	<0.001

### Dominance analysis

The results of the dominance analysis of four predictors (model 1, *P* = 4, Table 2) including resilience (X_1_), perceived social support (X_2_), self-efficacy (X_3_) and FOC (X_4_), showed that resilience, perceived social support, self-efficacy and FOC accounted for 33.0% (0.125/0.379), 18.7% (0.071/0.379), 19.0% (0.072/0.379) and 29.3% (0.111/0.379) of the total variance, respectively. A further dominance analysis (model 2, *P* = 3, Table 3) revealed that resilience (41.6% of the total variance) was the strongest predictor to psychological distress, followed by FOC (33.1% of the known variance) and self-efficacy (25.3% of the known variance). Thus, resilience, FOC and psychological distress were included in the following response surface analysis.

**TABLE 2:** When *P* = 4, the value-added contribution, average contribution and total average contribution of each predictor variable.

Variables (X)	*R* ^2^	Value-added contribution (Δ*R*^2^)			
		
		X_1_	X_2_	X_3_	X_4_
*K* = 0, average contribution	0	0.252	0.159	0.175	0.171
X_1_	0.252	–	0.039	0.023	0.082
X_2_	0.159	0.133	–	0.072	0.122
X_3_	0.175	0.100	0.056	–	0.121
X_4_	0.171	0.164	0.111	0.125	–
*K* = 1, average contribution	–	0.132	0.069	0.073	0.108
X_1_X_2_	0.292	–	–	0.010	0.076
X_1_X_3_	0.275	–	0.027	–	0.081
X_1_X_4_	0.334	—-	0.033	0.022	–
X_2_X_3_	0.231	0.071	–	–	0.104
X_2_X_4_	0.281	0.086	–	0.054	–
X_3_X_4_	0.296	0.061	0.040	–	–
*K* = 2, average contribution	–	0.073	0.033	0.029	0.087
X_1_X_2_X_3_	0.302	–	–	–	0.076
X_1_X_2_X_4_	0.367	–	–	0.011	–
X_1_X_3_X_4_	0.356	–	0.022	–	–
X_2_X_3_X_4_	0.335	0.043	–	–	–
*K* = 3, average contribution	–	0.043	0.022	0.011	0.076
X_1_X_2_X_3_X_4_	0.291	–	–	–	–
Total average contribution	–	0.125	0.071	0.072	0.111

X_1_: resilience, X_2_: perceived social support, X_3_: self-efficacy, X_4_: fear of childbirth.

**TABLE 3:** When *P* = 3, the value-added contribution, average contribution and total average contribution of each predictor variable.

Variables (X)	*R* ^2^	Value-added contribution (Δ*R*^2^)		
		
		X_1_	X_2_	X_3_
*K* = 0, average contribution	–	0.252	0.175	0.171
X_1_	0.252	–	0.023	0.082
X_2_	0.175	0.100	–	0.121
X_3_	0.171	0.164	0.125	–
*K* = 1, average contribution	–	0.132	0.074	0.102
X_1_X_2_	0.275	–	–	0.081
X_1_X_3_	0.334	–	0.022	–
X_2_X_3_	0.296	0.061	–	–
*K* = 2, average contribution	–	0.061	0.022	0.081
X_1_X_2_X_3_	0.356	–	–	–
Total average contribution	–	0.148	0.090	0.118

X_1_: resilience, X_2_: self-efficacy, X_3_: fear of childbirth.

### Response surface analysis

**FIGURE 1:**
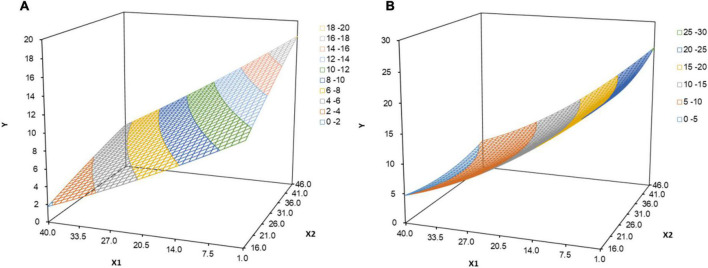
**(A)** In the absence of adverse childhood experiences, the effect of resilience and fear of childbirth on psychological distress. X_1_: resilience, X2: fear of childbirth, Y: psychological distress. **(B)** When having adverse childhood experiences, the effect of resilience and fear of childbirth on psychological distress. X_1_: resilience, X_2_: fear of childbirth, Y: psychological distress.

In the absence of ACEs, the result of the response surface analysis was presented in Figure 1A. Along the congruence line X = Y, psychological distress in the posterior corner (high FOC - high resilience) was significantly lower than that in the anterior corner (low FOC -low resilience), indicating that when the FOC was balanced with their resilience, pregnant women’s psychological distress level was lowest in the double-high combination. Along the discrepancy line X = –Y, psychological distress in the left corner of the graph (low FOC - high resilience) was significantly lower than those in the right corner of the graph (high FOC - low resilience), indicating that resilience had a more positive effect on psychological distress than the negative effect of FOC. A similar pattern emerged in pregnant women who experienced ACEs (Figure 1B). Besides, pregnant women with ACEs reported higher levels of psychological distress compared to those without ACEs.

### Mediation analysis

**FIGURE 2:**
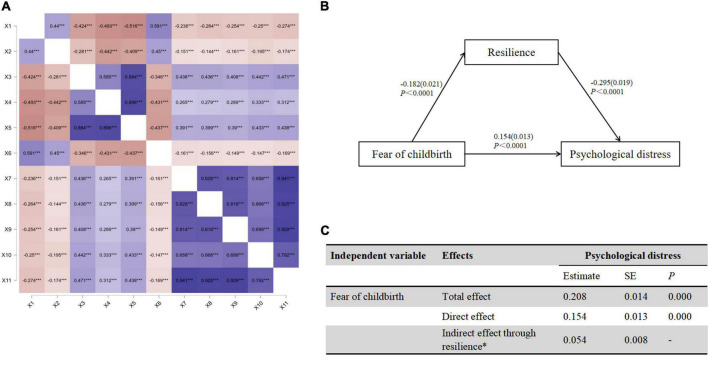
**(A)** Correlations between variables. **(B)** The mediating effect of resilience on psychological distress. **(C)** Direct and indirect (mediation) effect of resilience on psychological distress. X_1_: resilience, X_2_: perceived social support, X_3_: anxiety, X_4_: depression, X_5_: psychological distress, X_6_: self-efficacy, X_7_: baby-related FOC, X_8_: general and personal control-related FOC, X_9_: pain and injury-related FOC, X_10_: medical interventions and hospital care-related FOC, X_11_: Fear of childbirth. ***Correlation is significant at the 0.001 level.

**FIGURE 3:**
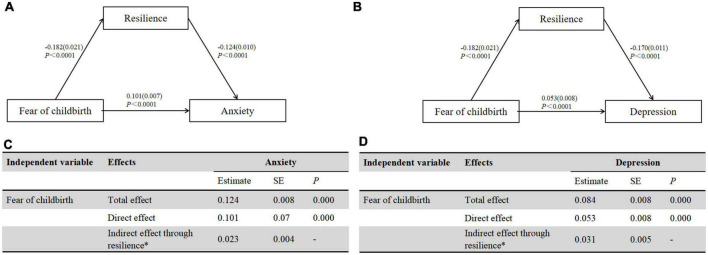
**(A)** The mediating effect of resilience on anxiety. **(B)** Direct and indirect (mediation) effect of resilience on anxiety. **(C)** The mediating effect of resilience on depression. **(D)** Direct and indirect (mediation) effect of resilience on depression.

The variance explained by the first factor was 27.6% and did not reach 50%, so the common method bias was negligible. Psychological distress was significantly correlated with resilience (*r* = –0.516, *P* < 0.001) and FOC (*r* = 0.438, *P* < 0.001). Other information was summarized in Figure 2A. Figure 2B revealed that FOC was negatively associated with resilience (β = –0.182, *P* < 0.001); resilience had a significant impact on anxiety (β = –0.124, *P* < 0.001, Figure 3A), depression (β = –0.170, *P* < 0.001) and psychological distress (β = –0.295, *P* < 0.001, Figure 3C). In Figure 2C, the indirect effect of FOC through resilience on psychological distress were significant (*B* = 0.054, SEBoot = 0.008, 95%CI: 0.08, 0.014), including anxiety (*B* = 0.023, SEBoot = 0.004, 95%CI: 0.016, 0.030, Figure 3B) and depression (*B* = 0.031, SEBoot = 0.005, 95%CI: 0.022, 0.041, Figure 3D).

### Moderated mediation analysis

**FIGURE 4:**
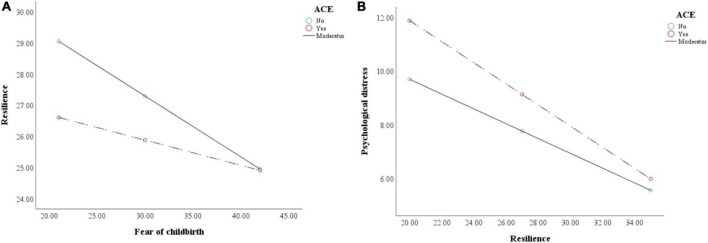
**(A)** The interaction between fear of childbirth and ace on resilience. **(B)** The interaction between resilience and ace on psychological distress.

The results of the moderation analysis were depicted in Tables 4–6. The interaction of FOC and ACEs was not significant (*B* = –0.036, 95%CI: –0.104 to 0.031, *P* = 0.291, Table 4), indicating that ACEs did not moderate the relationship between FOC and psychological distress. The significant moderation effect of ACEs was recognized (*B* = 0.114, 95%CI: –0.002 to 0.231, *P* = 0.054, Table 5) and visualized in the simple slopes test (B_no_ = –0.195, *P* < 0.001; B_yes_ = –0.084, *P* = 0.140, Figure 4A). In Table 6, ACEs could significantly moderate the association between resilience and psychological distress (B_no_ = –0.276, *P* < 0.001; B_yes_ = –0.393, *P* < 0.001, Figure 4B).

**TABLE 4:** The moderated effect of ACEs on the association between FOC and psychological distress.

Variables	*Estimate*	*SE*	*t*	*P*	LLCI	ULCI

Outcome variable: Psychological distress						
Constant	10.278	1.220	8.426	<0.001	7.884	12.671
Years	0.010	0.030	0.338	0.735	–0.049	0.069
Academic degree	–0.071	0.183	–0.391	0.696	–0.430	0.287
Marital status	0.952	0.593	1.606	0.109	–0.211	2.116
Working status	0.193	0.284	0.681	0.496	–0.363	0.750
Monthly average income	–0.024	0.299	–0.079	0.937	–0.610	0.563
Place of residence	0.250	0.283	0.883	0.378	–0.305	0.805
Maternity type	0.316	0.412	0.768	0.443	–0.492	1.125
Fear of childbirth	0.154	0.014	11.137	<0.001	0.127	0.182
Resilience	–0.289	0.018	–15.688	<0.001	–0.325	–0.253
ACE	2.722	1.193	2.281	0.023	0.381	5.063
Fear of childbirth × ACE	–0.036	0.034	–1.056	0.291	–0.104	0.031

**Increase of R^2^ with interaction**	***R* ^2^**		***F***		***P***	
	
	0.001		1.114		0.291	

**Conditional indirect effects of fear of childbirth on psychological distress**						

**ACE**	***Effect***	***SE***	***t***	***P***	**LLCI**	**ULCI**

No	0.154	0.014	11.137	<0.001	0.127	0.182
Yes	0.118	0.032	3.711	<0.001	0.026	0.181

**TABLE 5:** The moderated effect of ACEs on the association between FOC and resilience.

Variables	*Estimate*	*SE*	*t*	*P*	LLCI	ULCI

Outcome variable: Resilience						
Constant	30.589	1.866	16.392	<0.001	26.927	34.250
Years	0.074	0.052	1.425	0.155	–0.028	0.175
Academic degree	0.283	0.315	0.897	0.370	–0.335	0.900
Marital status	0.343	1.023	0.336	0.737	–1.664	2.350
Working status	–0.651	0.489	–1.333	0.183	–1.610	0.308
Monthly average income	0.973	0.515	1.890	0.059	–0.037	1.983
Place of residence	–0.430	0.488	–0.882	0.378	–1.387	0.527
Maternity type	–1.358	0.710	–1.914	0.056	–2.751	0.034
Fear of childbirth	–0.195	0.023	–8.454	<0.001	–0.241	–0.150
ACE	–4.845	2.052	–2.361	0.018	–8.872	–0.818
Fear of childbirth × ACE	0.114	0.059	1.930	0.054	–0.002	0.231

**Increase of R^2^ with interaction**	***R* ^2^**		***F***		***P***	
	
	0.003		3.724		0.054	

**Conditional effects of the focal predictor at values of the moderator (ACE)**						

ACE	***Effect***	***SE***	***t***	***P***	**LLCI**	**ULCI**

No	–0.195	0.023	–8.454	<0.001	–0.241	–0.150
Yes	–0.081	0.055	–1.476	0.140	–0.188	0.027

**Conditional indirect effects of fear of childbirth on psychological distress**						

**ACE**	***Effect***	***SE***	**LLCI**		**ULCI**	

No	0.058	0.009	0.041		0.076	
Yes	0.024	0.017	–0.009		0.058	

**TABLE 6:** The moderated effect of ACEs on the association between resilience and psychological distress.

Variables	*Estimate*	*SE*	*t*	*P*	LLCI	ULCI

Outcome variable: Psychological distress						
Constant	10.023	1.222	8.201	<0.001	7.625	12.421
Years	0.012	0.030	0.390	0.700	–0.047	0.070
Academic degree	–0.083	0.182	–0.457	0.648	–0.441	0.274
Marital status	0.867	0.592	1.465	0.143	–0.294	2.028
Working status	0.168	0.282	0.596	0.551	–0.386	0.722
Monthly average income	–0.001	0.298	–0.001	0.999	–0.586	0.585
Place of residence	0.247	0.2820	0.876	0.382	–0.307	0.801
Maternity type	0.295	0.411	0.719	0.472	–0.511	1.101
Fear of childbirth	0.150	0.013	11.704	<0.001	7.625	12.421
Resilience	–0.276	0.020	–14.127	<0.001	–0.314	–0.237
ACE	4.537	1.422	3.190	0.002	1.746	7.328
Resilience × ACE	–0.118	0.054	–2.189	0.029	–0.223	–0.012

**Increase of R^2^ with interaction**	***R* ^2^**		***F***		***P***	
	
	0.003		4.791		0.029	

**Conditional effects of the focal predictor at values of the moderator (ACE)**						

**ACE**	***Effect***	***SE***	***t***	***P***	**LLCI**	**ULCI**

No	–0.276	0.020	–14.127	<0.001	–0.314	–0.237
Yes	–0.393	0.051	–7.785	<0.001	–0.492	–0.294

**Conditional indirect effects of fear of childbirth on psychological distress**						

**ACE**	***Effect***	***SE***	**LLCI**		**ULCI**	

No	0.050	0.008	0.035		0.067	
Yes	0.072	0.015	0.045		0.103	

## Discussion

The current study examined the associations among FOC, resilience, ACEs and psychological distress in Chinese pregnant women. Specifically, we utilized dominance analysis, response surface analysis and moderated mediation model to test the effects of FOC, resilience, and ACEs on psychological distress.

First, FOC was positively associated with psychological distress in the current study, which was consistent with previous literature (58, 59). As FOC is defined as severe fear, anxiety or concern specifically in relation to childbirth (60), it may not be surprising that psychological distress was associated with higher FOC. However, the reasons for FOC are complex and unique and women with high to severe FOC is not recognized in maternity care in many countries. Hence, identifying pregnant women with high FOC may be the first step to help them build confidence in giving birth and improve their mental health.

Second, response surface results indicated that when resilience and FOC were congruent and high, pregnant women’s psychological distress level is significantly lower than the low resilience -low FOC model. In other words, a high level of FOC could be buffered by high resilience and pregnant women’s mental health will not be severely damaged. Furthermore, the effects of discrepancy in resilience and FOC on psychological distress suggested that low resilience – high FOC leads to higher psychological distress, while high resilience – low FOC promotes better mental health, indicating that resilience plays a significant role. These results were consistent with Huang’s findings (30) that pregnant women with high resilience may actively use their own psychological resources to reduce fear and ultimately maintain better mental health.

Third, the mediation model showed that resilience significantly mediated the relationship between FOC and psychological distress, which was partially confirmed in previous research (11). According to the pathway of FOC → resilience → psychological distress, it is essential that strategies to promote resilience in pregnant women need to be developed and implemented to counter the impact of FOC on psychological distress. There already exist resilience-enhancing programs for pregnant women. For instance, Witteveen et al. developed a guided self-help ACT-based program for pregnant and the psychological distress symptoms were alleviated (61). In addition, resilience-based interventions have also been trialed on patients. For example, Ye et al. developed the program Be Resilient to Breast Cancer to promote breast cancer patients’ resilience, resulting in increased quality of life and hope (62–68). These successful programs could be adapted and utilized for pregnant women.

Fourth, ACEs was confirmed as a moderator among FOC, resilience and psychological distress. On the one hand, FOC had a stronger negative effect on resilience in pregnant women with ACEs compared to those without ACEs, indicating that ACEs contributed to reduced resilience, which was consistent with prior studies (69–71). On the other hand, when pregnant women have experienced childhood trauma, the protective effects of resilience are diminished, leading to them more prone to psychological distress. Our findings support the growing literature acknowledging the long-term and severe impact of ACEs on psychological distress (72, 73). So, it is crucial to incorporate ACEs screening into prenatal care.

In general, pregnant women with ACEs, high FOC and low resilience are more likely to experience severe psychological distress, which should be addressed through early identification and intervention.

### Limitations

The present study has some limitations. First, all the collected data is self-reported, so recall bias and social desirability bias may exist. Second, conclusions derived from this study are based on Chinese pregnant women and these findings should be replicated in other populations with different backgrounds. Third, the cross-sectional design limits the ability for causal inference among these four variables, and a longitudinal study should be conducted to replicate these findings. An ongoing 2 year follow-up assessment of this cohort (Be Resilient to Postpartum Depression, BRPD) will provide additional insights in the future. Fourth, several potential confounders, such as intimate partner violence, sleep quality and mindfulness level are not considered in the moderated mediation model due to heavy scale burden, which may result in biased estimations of associations.

## Conclusion

Fear of childbirth (FOC) directly impacts psychological distress, and resilience significantly mediates the relationship between FOC and psychological distress. ACEs significantly moderates the association between FOC and psychological distress as well as the relationship between resilience and psychological distress. Reducing FOC while promoting resilience and early screening of ACEs may be useful targets for relieving psychological distress level among pregnant women in China.

## Data availability statement

The data that support the findings of this study are available on request from the corresponding authors. The data are not publicly available due to privacy or ethical restrictions.

## Ethics statement

The studies involving human participants were reviewed and approved by the Ethics Committee of the First Affiliated Hospital of Guangzhou University of Chinese Medicine (No. K-2022-024). The patients/participants provided their written informed consent to participate in this study.

## Author contributions

XM: conceptualization, data curation, methodology, software, and writing – original draft. RM: software, validation, and methodology. YLL: investigation, software, and methodology. XW and QC: investigation and resources. YJL: resources and supervision. ZY: supervision and writing – review and editing. All authors contributed to the article and approved the submitted version.
